# Annotations

**Published:** 1902-12-27

**Authors:** 


### ANNOTATIONS.

### The Multiplication of Universities.

THE inquiry into the petition of the Liverpool University College for a charter for a separate university, which has lately taken place at the Privy Council Office, practically terminated last Friday, the Duke of Devonshire announcing that in the opinion of the committee they had sufficient before them to enable them to make a report. The matter to be dealt with is one of great importance, for it can hardly be doubted that if university status should be granted to the Liverpool College, the disruption of the Victoria University and the establishment of three separate universities in its place, one in Liverpool, one in Manchester, and one in Leeds, must rapidly follow. Thus the affair touches interests which extend far beyond the districts which are more particularly concerned in the northern university, of which the petitioning body is a constituent college. Moreover, it is a matter which is of especial importance to the medical profession at large. That the multiplication of universities competing against one another for the patronage of fee-producing students must tend to the degradation of university degrees, goes almost without saying; and even if these degrees were merely decorations and hall-marks, proof and promise of good work, any cheapening of such insignia would be a thing to be regretted. But medical degrees are something much more than this. They stand upon a totally different footing from the rest, in that they give admission to the Medical Register and thus are a legal qualification for medical practice. Thus it is that in the maintenance of the educational standard required of medical graduates the public, as well as the medical profession, have a far greater interest than they have in the status of mere graduates in arts. It is because each of these new single College Universities becomes yet one more mode of entry to a profession into which there are already so many competing portals, and because if they are to live they must enter with the rest into the general scramble for students which is now going on, that their multiplication becomes a matter of such serious interest.

### "Sitting on the Pill."

THE recipe for a patent pill was sold a short time ago for £5,000. The auctioneer, in recommending it to possible purchasers, dilated on the advantages of owning a patent medicine. It required no offices, he said, no expensive plant, and apparently no costly drugs; as he put it, you have simply to "stick a cigar in your mouth and sit upon the pill," and you are sure of a comfortable, and even luxurious living. The pill in question professes to be compounded of quinine and dandelion, drugs which it is in the power of any doctor or chemist to obtain. No great skill is wanted in compounding a pill, and yet how many medical men are there who fail to obtain by hard and conscientious work the £1,000 a year which it is stated this pill brought in to its lucky possessor. But the auctioneer who successfully disposed of this magic prescription omitted one important ingredient in its success—the credulity of the drug-swallowing public. One might also add that the purchaser can hardly sit down on the pill, or on any easy-chair provided for it. He must run about the country, either personally or by agent, pushing the pill before him, singing or shouting its praises, and above all gathering up testimonials in its favour. It would be interesting to know how all the testimonials in favour of all the quack medicines before the public have been obtained. There must be an endless number of men and women in the back streets of our cities—always the back streets—who have "suffered agonies for years," and been "given up by the doctors"—what doctors we wonder—until the magic medicine was brought before their notice. If one-half of the symptoms which they detail are true, they ought by all the laws of pathology to have been dead years before they came across it. But they survive for the sole object, as it would seem, of vaunting the merits of "Jones' Syrup," or "Tomkins' Pill."

### "Tubes" *versus* "Points."

THE *Medical Record* justly says that the story of the manner in which small-pox was cleared out of Porto Rico furnishes "a lesson to the world." When the Spaniards left the island in 1898, small-pox was already endemic in the island, and it spread so rapidly that it soon became necessary to make a vigorous effort for its suppression. With this object compulsory vaccination was brought to play, and so thoroughly was it enforced that in four months in a population of 960,000 no fewer than 860,000 vaccinations were performed. The work then ceased, the disease having practically disappeared, and this, notwithstanding the numerous centres of infection which existed, the indifference of the people, the hiding of the cases, the absolute impossibility of securing isolation in the crowded dwellings, and the great insufficiency of the medical help which was available. From a medical point of view, the greatest interest in this experience lies not in its success, which, indeed, was a foregone conclusion, but in the strong opinion expressed in the report on the subject as to the failure of lymph preserved in tubes, and as to the advantages of reverting again to the old-fashioned ivory "points", at any rate in hot climates. It was conclusively demonstrated, we are told, that the lymph when glycerinated and preserved in tubes did not retain its efficiency when exposed even briefly to the change from a temperate to a tropical climate, and nothing was found so satisfactory as the ivory "point," whether for taking virus or for vaccinating animals or people. This is a matter of considerable interest. Of course we all accept the guarantees, such as they are, which are given by glycerinated calf-lymph as a great advance; yet there are plenty of practitioners now living who look back with a feeling of regret to the old days of "points," and to the time when by a judicious selection of cases and a careful collection of lymph under his own eye a man might attain a certain degree of local repute for the perfection of his vaccination, and many are ready to compare the customs of those days distinctly favourably with those of the present time, when a vaccinator can but employ the stuff that is sent him—stuff prepared in some unknown laboratory by some unknown person, and as to the purity of which both he and the patient have to trust to the label on the packet.

